# Adrenocorticotropic Hormone Secreting Pheochromocytoma Underlying Glucocorticoid Induced Pheochromocytoma Crisis

**DOI:** 10.1155/2018/3963274

**Published:** 2018-02-20

**Authors:** Gil A. Geva, David J. Gross, Haggi Mazeh, Karine Atlan, Iddo Z. Ben-Dov, Matan Fischer

**Affiliations:** ^1^The Hebrew University Hadassah Medical School, Hadassah-Hebrew University Medical Center, Jerusalem, Israel; ^2^Endocrinology & Metabolism Service, Hadassah-Hebrew University Medical Center, Jerusalem, Israel; ^3^Department of General Surgery, Hadassah-Hebrew University Medical Center, Jerusalem, Israel; ^4^Department of Pathology, Hadassah-Hebrew University Medical Center, Jerusalem, Israel; ^5^Nephrology and Hypertension Services, Hadassah-Hebrew University Medical Center, Jerusalem, Israel; ^6^Department of Internal Medicine, Hadassah-Hebrew University Medical Center, Jerusalem, Israel

## Abstract

**Context:**

Pheochromocytomas are hormone secreting tumors of the medulla of the adrenal glands found in 0.1–0.5% of patients with hypertension. The vast majority of pheochromocytomas secrete catecholamines, but they have been occasionally shown to also secrete interleukins, calcitonin, testosterone, and in rare cases adrenocorticotropic hormone. Pheochromocytoma crisis is a life threatening event in which high levels of catecholamines cause a systemic reaction leading to organ failure.

**Case Description:**

A 70-year-old man was admitted with acute myocardial ischemia following glucocorticoid administration as part of an endocrine workup for an adrenal mass. Cardiac catheterization disclosed patent coronary arteries and he was discharged. A year later he returned with similar angina-like chest pain. During hospitalization, he suffered additional events of chest pain, shortness of breath, and palpitations following administration of glucocorticoids as preparation for intravenous contrast administration. Throughout his admission, the patient demonstrated both signs of Cushing's syndrome and high catecholamine levels. Following stabilization of vital parameters and serum electrolytes, the adrenal mass was resected surgically and was found to harbor an adrenocorticotropic hormone secreting pheochromocytoma. This is the first documented case of adrenocorticotropic hormone secreting pheochromocytoma complicated by glucocorticoid induced pheochromocytoma crisis.

**Conclusion:**

Care should be taken when administering high doses of glucocorticoids to patients with suspected pheochromocytoma, even in a patient with concomitant Cushing's syndrome.

## 1. Introduction

Pheochromocytomas are a group of hormone secreting tumors that arise from chromaffin cells in the medulla of the adrenal glands. Pheochromocytoma manifests with an array of clinical symptoms including headaches, sweating, palpitations, and hypertension. The prevalence of pheochromocytoma in patients diagnosed with hypertension is 0.1–0.5% [1]. Pheochromocytomas are usually functional and secrete catecholamines. In rare cases they have been shown to also secrete interleukins, calcitonin, and testosterone [2].

Adrenocorticotrophic hormone (ACTH) is a 39-amino acid pituitary hormone which promotes adrenal hyperplasia and glucocorticoid synthesis in response to physiological stress. Ectopic ACTH secretion accounts for 10–20% of ACTH-dependent Cushing syndrome and mostly originates from bronchial or thymic neuroendocrine tumors or small cell lung carcinomas.

We report a case of a 70-year-old man, who presented with recurrent episodes of chest pain and hypertension refractory to treatment, following glucocorticoid administration. He was found to have an ACTH secreting pheochromocytoma.

## 2. Case Report

A 70-year-old man presented to the emergency department at our institution, with angina-like chest pain, palpitations, and sweating.

One year prior to his current admission, he underwent endocrine evaluation following an incidental adrenal finding on imaging measuring 25 × 34 mm^2^. A 24-hour urine collection for catecholamines revealed a urine epinephrine level of 150 *μ*g (normal limit: <27 *μ*g/day). A 1-mg overnight dexamethasone suppression test was abnormal, with serum cortisol level of 188 nmol/l (normal limit < 50 nmol/l). He then underwent an ambulatory high dose dexamethasone suppression test. That day he was referred to the emergency department due to angina-like chest pain, palpitations, and sweating. He underwent cardiac catheterization, which demonstrated no significant pathology in the coronary arteries, and was discharged the next day. Past medical history was significant for hypertension, type II diabetes mellitus, and dyslipidemia.

A year later, on current admission, the patient's electrocardiogram showed sinus rhythm, new T wave inversion in lateral and posterior leads, with no conduction abnormalities. Bedside echocardiography demonstrated hypokinesis in the distribution of the left anterior descending coronary artery. The patient underwent urgent catheterization which once again demonstrated no pathology in the coronary arteries. A subsequent echocardiogram demonstrated normal ventricular function with moderate mitral and tricuspid regurgitation.

During his stay in the coronary care unit, the patient experienced several episodes of hypertension and tachycardia, refractory to treatment with calcium channel blockers, beta and alpha adrenergic blockade, angiotensin receptor blockers, and furosemide. As part of resistant hypertension evaluation, abdominal computed tomography was performed and a 36 × 34 × 22 mm^3^ right adrenal mass was identified. The adrenal mass had a density of 39 Hounsfield units (HU) prior to intravenous contrast injection, increasing to 74 HU following contrast injection. Notably, the patient experienced a severe event of hypertension, palpitations, and chest pain following administration of 100 mg hydrocortisone as preparation for contrast agent infusion as he had received a diagnosis of sensitivity to intravenous contrast material. Plasma metanephrine was found to be 1200 pg/ml (normal < 90 pg/ml) with a normal normetanephrine level of 163 pg/ml (normal < 196 pg/ml). Plasma cortisol level at 8 am, after overnight 1-mg dexamethasone suppression, was 2770 nmol/l, while level at 8 am without dexamethasone suppression was 3292 nmol/l (normal range 100–690 nmol/l). ACTH level was 174 pmol/l (normal range 1.9–10.2 pmol/l). Renin and aldosterone levels were within normal limits.

The patient's case was presented at a multidisciplinary team meeting and surgery was advised. Following 2 weeks of alpha blockade, uneventful laparoscopic right adrenalectomy was performed, with stable blood pressure throughout the procedure. The postoperative course was uncomplicated and the patient was discharged on day two.

**Figure 1:**
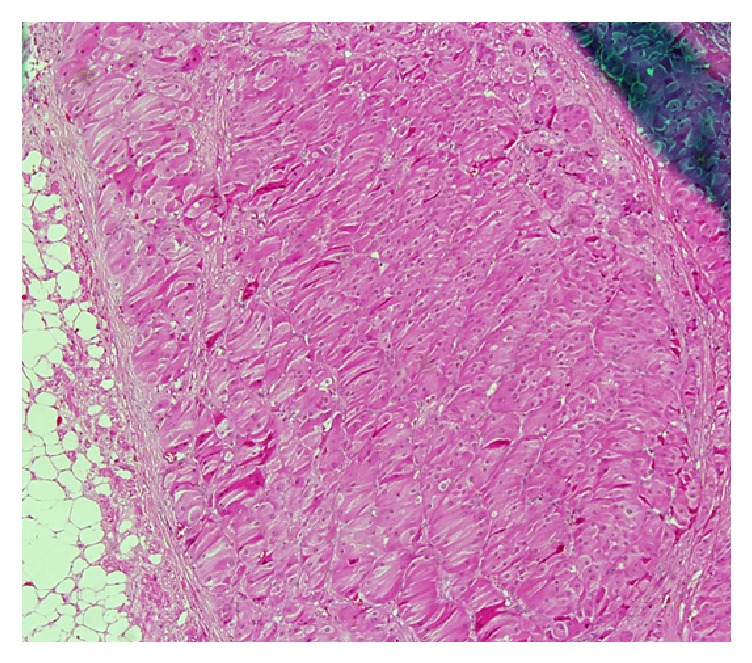
Hyperplastic adrenal cortex.

**Figure 2:**
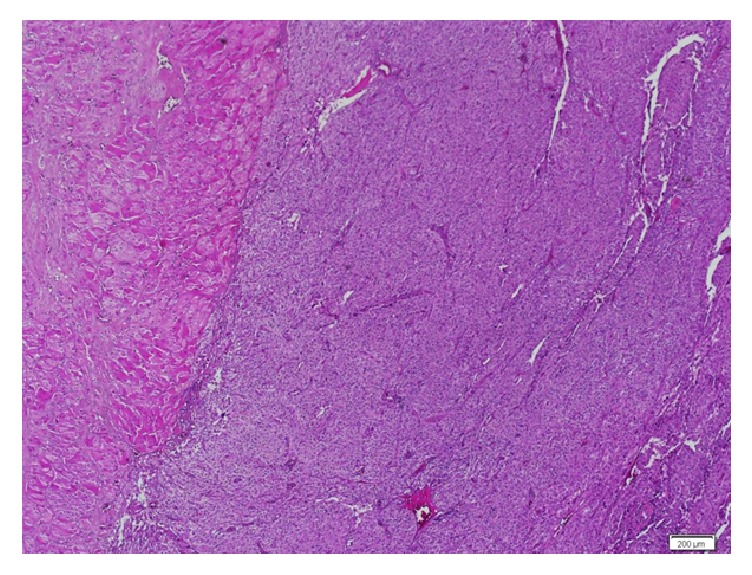
Adrenal gland, pheochromocytoma, and diffuse growth pattern with high cellularity. H&E ×40.

**Figure 3:**
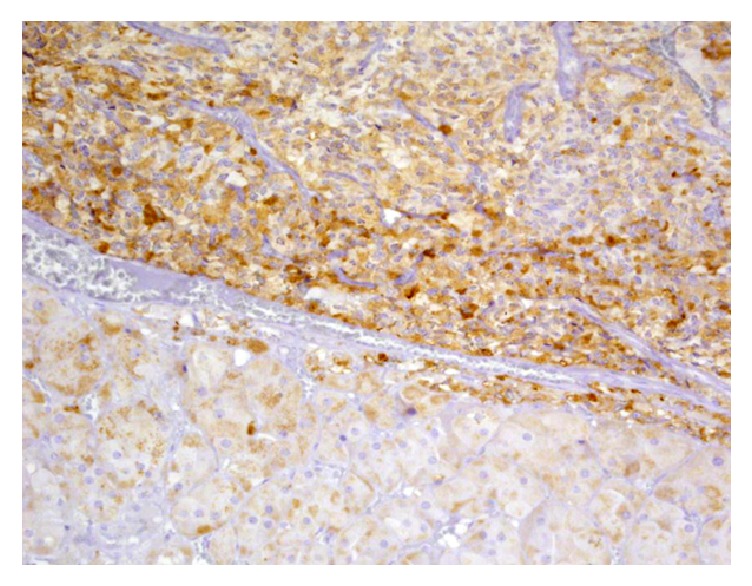
Positive Immunostaining for ACTH compatible with ectopic ACTH secretion by the tumor.

**Figure 4:**
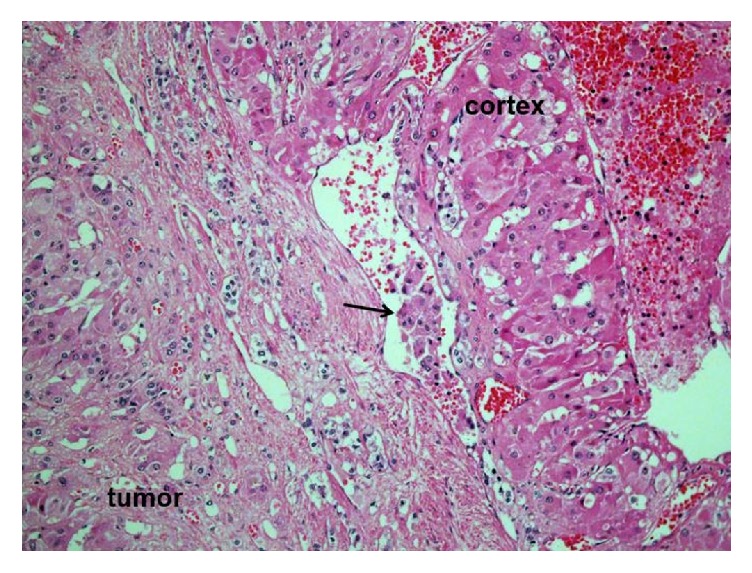
Tumor, vascular invasion at tumor edge, and hyperplastic cortex. The arrow represents vascular invasion of the tumor.

Pathology revealed a 36 mm pheochromocytoma of the adrenal gland with a scaled score (PASS) of 7 (vascular invasion, predominantly diffuse growth, high cellularity, and spindling of cells). Diffuse adrenal cortical hyperplasia was noted (Figure 1). Immunostaining was positive for ACTH and synaptophysin and negative for chromogranin, inhibin, calretinin, and S-100 protein.  Figure 2 shows the pathology of adrenal tumor, pheochromocytoma, and diffuse growth pattern.  Figure 3 depicts positive immunostraining for ACTH.  Figure 4 depicts the tumor's vascular invasion, and the adrenal gland's hyperplastic cortex.

During 23 months of follow-up the patient had no cardiac events, his blood pressure decreased to 126/79, and he was able to decrease his antihypertensive medications. A CT scan performed 7 months following surgery revealed normal postoperative changes with no evidence of recurrence.

## 3. Discussion

Cushing's syndrome was first described in 1912 by Cushing [3]. The syndrome, which is caused by chronic exposure to abnormally high levels of the stress hormone cortisol, may present with a variety of clinical symptoms, none of which is sensitive or specific. The common manifestations include hypertension, diabetes mellitus, central obesity, proximal muscle wasting and weakness, hirsutism, red-purple striae, and oligomenorrhea in women [4].

Excess ACTH accounts for 80% of cases, while the remaining 20% are ACTH-independent. Of the ACTH-dependent Cushing syndrome cases, 80%–90% are due to Cushing's disease, pituitary corticotroph adenoma [5], and 10%–20% are due to ectopic ACTH secreting tumors. Although ectopic ACTH secreting tumors are most commonly bronchial carcinoid, thymic carcinoid, or small cell lung cancer [6], more than 2 dozen cases of ACTH secreting pheochromocytomas have been described in the literature [2, 6–10].

Pheochromocytoma crisis (PC) is a potentially life threatening event caused by high levels of catecholamines secreted by the neoplastic chromaffin cells leading to organ failure [11, 12]. While many drugs have been shown to cause PC, reports of glucocorticoid induced PC are rare and mostly limited to single case reports [9, 13–17].

Our patient experienced his first event of refractory hypertension and angina-like chest pain severe enough to warrant cardiac catheterization following administration of high dose dexamethasone. The second substantial event occurred several hours after glucocorticoid administration as preparation for contrast agent infusion due to intravenous contrast sensitivity. Glucocorticoids play a fundamental part in catecholamine metabolism, production, and release both in the healthy adrenal medulla and in pheochromocytoma cells. Glucocorticoids were shown to induce, in a dose dependent manner, enzymes required for catecholamine synthesis, including phenylethanolamine-N-methyltransferase, which converts norepinephrine to epinephrine, tyrosine hydroxylase, a rate-limiting enzyme in catecholamine metabolism, and proopiomelanocortin, an ACTH precursor [9, 13].

While a normal adrenal medulla would not be greatly affected by exogenous glucocorticoids, the loss of anatomical and cellular barriers in pheochromocytoma may increase the susceptibility of the chromaffin cells to glucocorticoids.

To the best of our knowledge this is the only published report of an ACTH secreting pheochromocytoma underlying a glucocorticoid induced PC. It is possible that Cushing's syndrome background of our patient increased his susceptibility to PC as he was constantly exposed to high levels of glucocorticoids. None of the reports regarding ACTH secreting pheochromocytoma mention a clinical state of PC, but as both these conditions are extremely rare further data are required to establish whether a pheochromocytoma with Cushing's syndrome is more likely to be complicated with pheochromocytoma crisis.

In a review of the literature, Rosas et al. [13] present 11 cases of glucocorticoid induced PC. Of those cases at least two experienced a pheochromocytoma crisis following high dose dexamethasone suppression. Given the significant risk for morbidity and mortality of pheochromocytoma crisis and the unpredictability of PC following glucocorticoid treatment, we suggest caution when administering glucocorticoids to patients with suspected pheochromocytoma.
